# Application of artificial intelligence in life science: Historical review and future perspectives

**DOI:** 10.1016/j.fmre.2024.10.014

**Published:** 2024-11-08

**Authors:** Lei Shi, Meng Wang, Xiu-Jie Wang

**Affiliations:** a Institute of Genetics and Developmental Biology, Chinese Academy of Sciences, Beijing 100101, China; b School of Future Technology, University of Chinese Academy of Sciences, Beijing 100049, China

**Keywords:** Artificial intelligence, Bioinformatics, Bibliometrics, Life science research, Algorithm

## Abstract

The fast advancement of artificial intelligence (AI) technologies in recent years, especially the deep learning algorithm and transformer neural network, has brought great impacts on scientific researches. As a displine focusing on curation and analysis of life science data, bioinformatics has experienced remarkable changes under the impetus of AI technologies, not only in nowadays, but also throughout its history. In this review, we firstly summarize the historical events of computer-assisted life science data analysis, then assess the features, contributions and changes of AI methods in life science research by using bibliometric analysis, and discuss the future challenges for AI methods from the life science research aspects. There is no doubt that AI technologies will continuously accelerate and revolutionize life science research in the future, in the meanwhile, the development of new AI methods more suitable for life science data is also in great needs.

## 1 Introduction

**Fig. 1:**
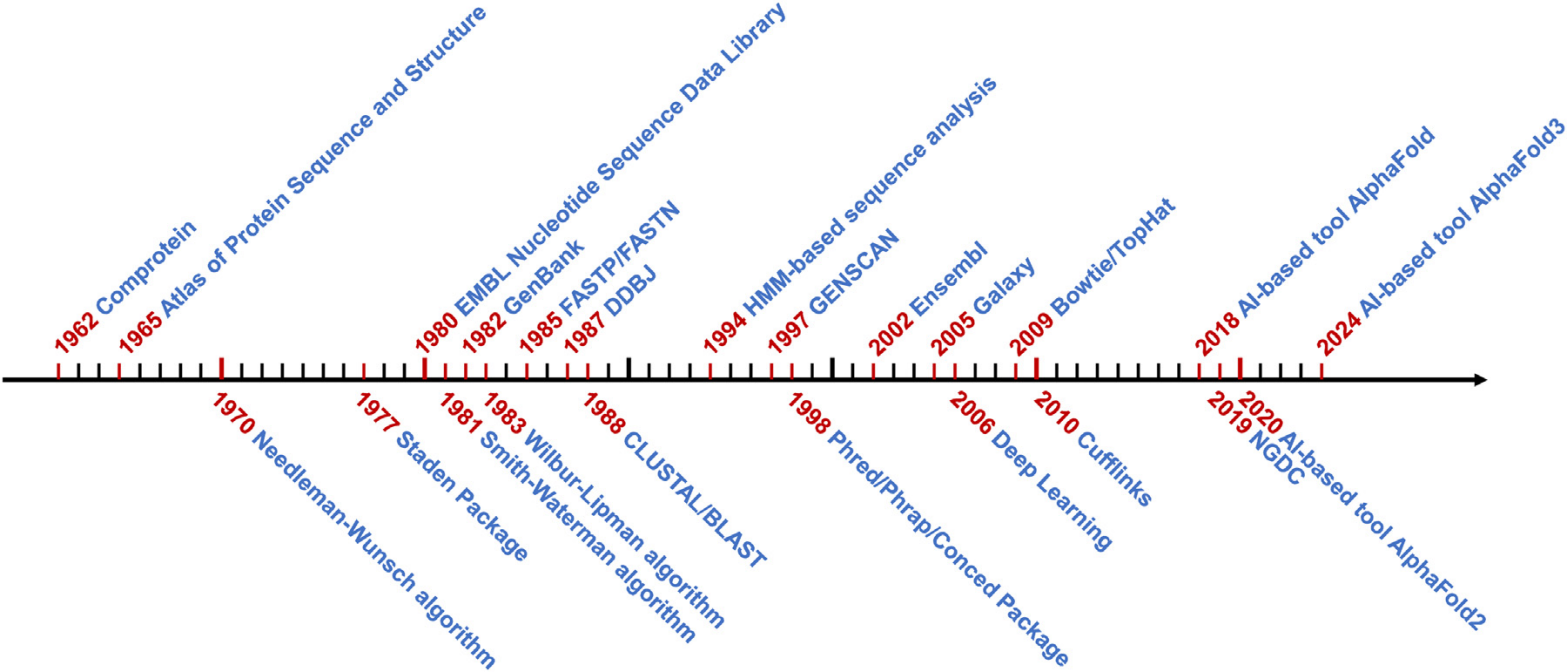
**The historical events of Bioinformatics.** The black arrow represents the timeline, with ticks represent continuous years starting from 1962. Representative algorithms, databases and software with significant impacts in the history of bioinformatics are displayed, with corresponding years highlighted in red.

Bioinformatics, as a cutting-edge discipline to curate, integrate, analyze and decipher biological and biomedical data, is becoming increasingly important for life science research, especially with the broad application of various omics-based techniques in recent years. Besides the rapid accumulation of biological data, another indispensable driving force for bioinformatics is the rapid development of computer science, especially artificial intelligence (AI) related data analysis methods. Here, we will briefly review the history of bioinformatics (Fig. 1), analyze AI-related life science publications from different aspects, and discuss the future trends of AI-assisted life science research.

## 2 The historical events of computer-assisted life science data analysis

Although the word “Bioinformatics” was first coined in the late 1980s [1], the application of computer in life science research can be dated back to the 1950s. At that time, protein was the major research focus of life science, and scientists started to sequence proteins using methods developed by Frederick Sanger or Pehr V. Edman [[2], [3], [4], [5], [6], [7]]. To derive the amino acid composition of proteins from the sequencing results, Margaret O. Dayhoff and Robert S. Ledley developed the first bioinformatics software, Comprotein [8], which was written in Fortran language and run on an IBM7090 mainframe computer. In 1965, Margaret O. Dayhoff and colleagues compiled the sequences of 65 proteins and built the first biological sequence database, the Atlas of Protein Sequence and Structure, which was released as a 95-page printed book [9]. To facilitate computer analysis, Comprotein for the first time used three alphabet abbreviations to represent each amino acid, which later evolved into the single-letter amino acid code. Such amino acid abbreviations are still used today and serve as the basis for protein sequence alignment and comparison.

In 1970, Saul B. Needleman and Christian D. Wunsch for the first time applied dynamic programming, a popular algorithm in AI, on protein sequence analysis, and developed the Needleman-Wunsch algorithm for global alignment of two protein sequences [10]. Based on this, Temple F. Smith and Michael S. Waterman developed the Smith-Waterman algorithm for local protein sequence alignment in 1981 [11]. In 1987, Da-Fei Feng and Russell F. Doolittle developed the first practical multiple protein sequence alignment and phylogenetic tree construction method [12]. One year later, Desmond G. Higgins and Paul M. Sharp developed the CLUSTAL method [13], which is still widely used for multiple sequence alignment and sequence phylogenetic tree construction today.

Soon after Frederick Sanger developed the DNA sequencing technology in 1977, Roger V. Staden developed the Staden Package [14], a software for analyzing Sanger sequencing results. Staden Package is considered as a pioneer bioinformatics analysis software, which contains a complete set of tools for DNA sequence assembly, editing, and analysis. In 1980, the first nucleic acid sequence database, EMBL Nucleotide Sequence Data Library (now known as the European Nucleotide Archive, ENA), was established at the European Molecular Biology Laboratory in Heidelberg, Germany. Two years later, GenBank was established by the National Institutes of Health of the USA, which later became the most influential genetic sequence database in the world. In 2016, the Beijing Institute of Genomics under Chinese Academy of Sciences founded the BIG Data Center (BIGD) [15], which was renamed as National Genomics Data Center (NGDC) in 2019. NGDC is the first national biological data center in China. It contains various types of sequence resources as well as a full collection of bioinformatics analysis tools, and is now the central data hub of China National Center for Bioinformation (CNCB).

In 1980s, the management and analysis of life science data have greatly benefited from the development of computer science, especially the improvement of data processing capabilities and the introduction of new machine learning algorithms. In 1983, W. John Wilbur and David J. Lipman developed the Wilbur-Lipman algorithm for sequence search in databases [16], which performed global sequence comparison based on matching k-tuples of sequence elements for a given k. In 1985, the first fast sequence similarity search tool, FASTP [17], was developed, followed by the release of the famous sequence similarity search tool BLAST in 1990 [18]. With these progresses, bioinformatics has emerged a new discipline.

Since the mid-1990s, the development of microarray and parallel sequencing technologies have revolutionized the paradigm of life science research. Multiple types of microarray platforms have been invented and applied on genotyping, transcriptional profiling, DNA methylation detection, protein interaction analysis, disease marker screen, and other scientific or clinical questions. Parallel sequencing technologies, from the early days Serial Analysis of Gene Expression (SAGE) and Massive Parallel Signature Sequencing (MPSS) technologies, to nowadays widely used next generation sequencing and third generation sequencing technologies, as well as single-cell and spatial omics technologies, have become indispensable approaches for biological and biomedical researches. A huge amount of sequencing data has been generated, which in turn promoted the development of biological databases and data analysis software. Many AI algorithms, such as decision tree [19], hidden Markov model [20], principal component analysis [21], random forest [22], support vector machine [23,24], and others, have been widely applied in life science data analysis. In recent years, the revolutionized advances of new AI methods, such as deep learning [[25], [26], [27]], large language model [28], transformer neural network [29] and generative AI [30], are expected to lead life science research into a new era.

## 3 Overview of AI related life science research papers

To systematically analyze the application of AI methods in life science research, we collected all literature published before 2024 in the PubMed database, and selected the ones with MeSH term matching “Artificial Intelligence”. A total of 185,795 qualified papers were collected, after filtering out papers with missing information, 139,799 papers were kept for further analysis.

**Fig. 2:**
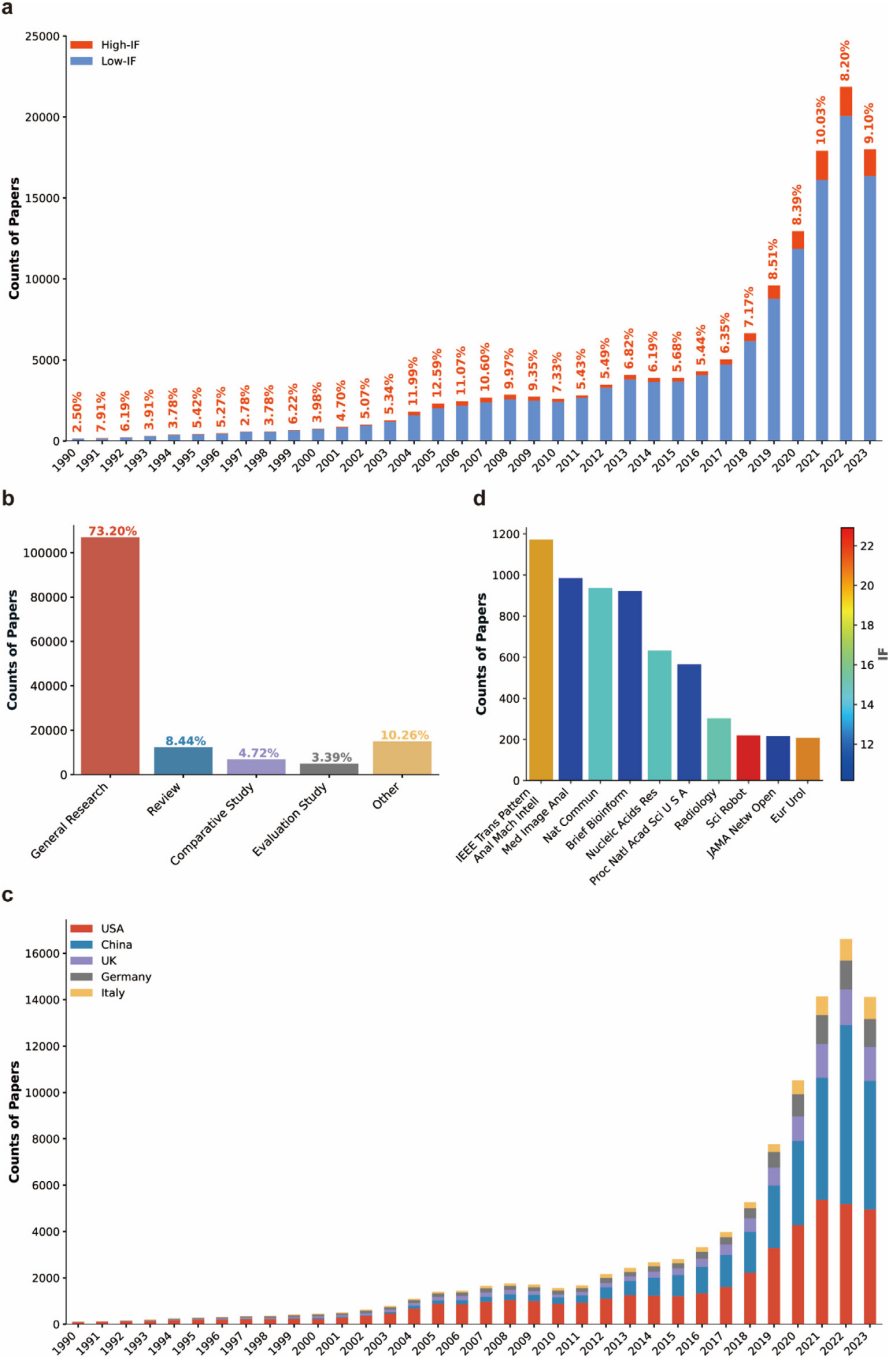
**Overview of AI-related life science research papers**. (a) Annual counts of AI-related life science research papers since 1990. High-IF and Low-IF represent papers published in journals with IF ≥ 10 and IF < 10, respectively. Numbers shown on the top of each bar represent the proportion of high-impact papers published in the corresponding year. (b) Publication type analysis of AI-related life science research papers. Numbers on each bar represent the ratio of the corresponding publication type among all examined papers. (c) Annual comparison of AI-related life science research papers among the top 5 countries ranked by total publication counts. (d) The top 10 journals ranked by publication counts of AI-related life science research papers.

Among the 139,799 papers, only 867 papers were published before 1990. Therefore, in the follow up analysis, we solely focused on papers published from 1990 and onwards. The analysis results showed that the annually published AI-related life science papers increased gradually, from 160 in 1990 to 17,998 in 2023, with a burst after 2018 (Fig. 2a). It is worth to note that the proportion of AI-related life science literature published in high-impact journals experienced two waves. The first wave was from 2004 to 2009, in accordance with the fast development and application of the second-generation sequencing technologies. The second wave started since 2018, probably benefited from the advancement of AI technologies, especially the application of the deep learning algorithm and large language models, as exemplified by the development of AlphaFold in 2018 and the later release of AlphaFold2 in 2020, followed by AlphaFold3 in 2024 [[31], [32], [33], [34]]. The inventors of AlphaFold2, Demis Hassabis and John Jumper, have been awarded the Nobel Prize in Chemistry in 2024. Paper type analysis revealed that 73.20% of AI-related life science literature are general research papers, 8.44% are reviews, followed by comparative studies (4.72%) and evaluation studies (3.39%), and the rest types of papers counted for 10.26% in total (Fig. 2b).

Internationally, the USA leads the AI-related life science with 43,137 publications since 1990. More importantly, among the papers published before 1990, 51.29% were published by American universities or research institutions. Until 2013, more than half of the AI-related life science papers in PubMed were published by research groups in USA. China started AI-related life science research in 1990s, and the related papers increased rapidly since 2011, with a burst after 2018 (Fig. 2c). From 1990 to 2023, China has published 33,989 AI-related life science papers. In 2022 and 2023, the number of AI-related life science papers published by Chinese researchers had surpassed that by US researchers. The UK ranked the third in terms of published AI-related life science papers from 1990 to 2023, followed by Germany and Italy, with a total of 10,625, 9,181 and 6,052 published papers, respectively (Fig. 2c).

In terms of scientific journals, IEEE Transactions on Pattern Analysis and Machine Intelligence (IEEE Trans Pattern Anal Mach Intell) published the largest amount of AI-related life science papers by the end of 2023, followed by Medical Image Analysis (Med. Image Anal.), Nature Communications (Nat. Commun.), and Briefings in Bioinformatics (Brief Bioinform.) (Fig. 2d). Other journals ranked the top 10 in terms of published AI-related life science papers are shown in Fig. 2d.

## 4 Importance of collaborations in AI-related life science research

**Fig. 3:**
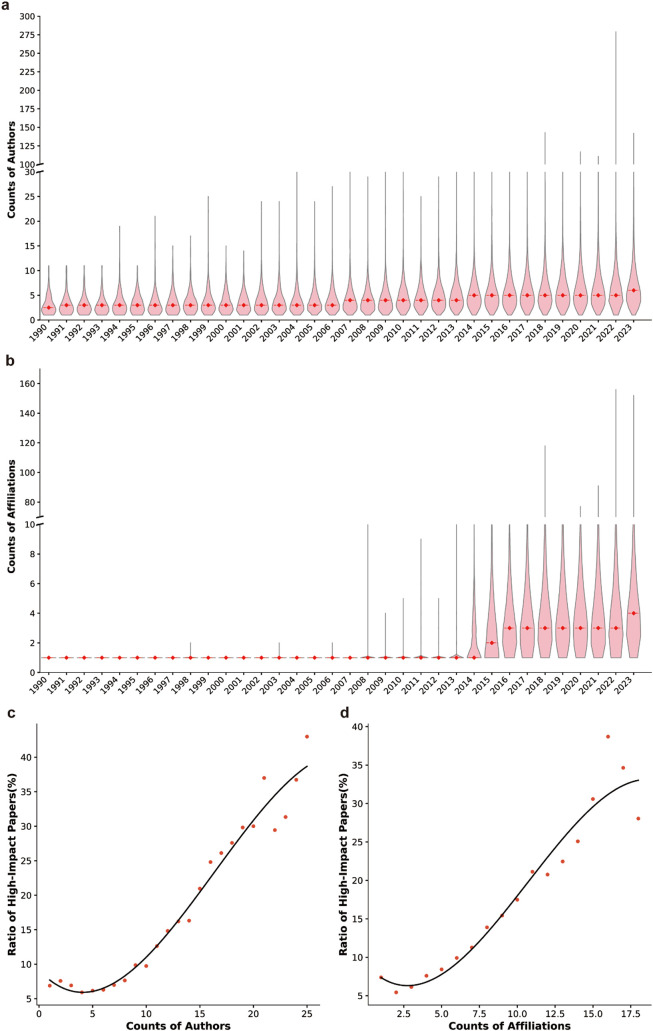
**Collaboration effect analysis on AI-related life science research papers**. (a-b) Annual analysis of author (a) or affiliation (b) numbers per article. Violin plots show the distribution of author or affiliation counts per article in each year, and box plots show the quartile distribution of author or affiliation counts, with the bottom, middle and upper lines of the boxes show the first quartile (Q1), median, and third quartile (Q3) of the author or affiliation counts per article, respectively. (c-d) Correlations between the number of authors (c) or affiliations (d) per article and the impact of publications. High-impact papers represent papers published in journals with IF ≥ 10.

We next counted the number of authors and affiliations per paper for collaboration analysis. The results revealed that although the average number of authors of these AI-related life science papers showed an overall trend of increment (Fig. 3a), multi-institutional collaborations only became dominant after 2014 (Fig. 3b). When evaluating the quality of AI-related life science papers solely by the impact factors of their published journals, a clear correlation was observed between the number of authors or author affiliations and the impact of paper published journals (Fig. 3c, 3d), demonstrating the importance of large scale collaborations in generating high-impact publications in AI-related life science research.

**Fig. 4:**
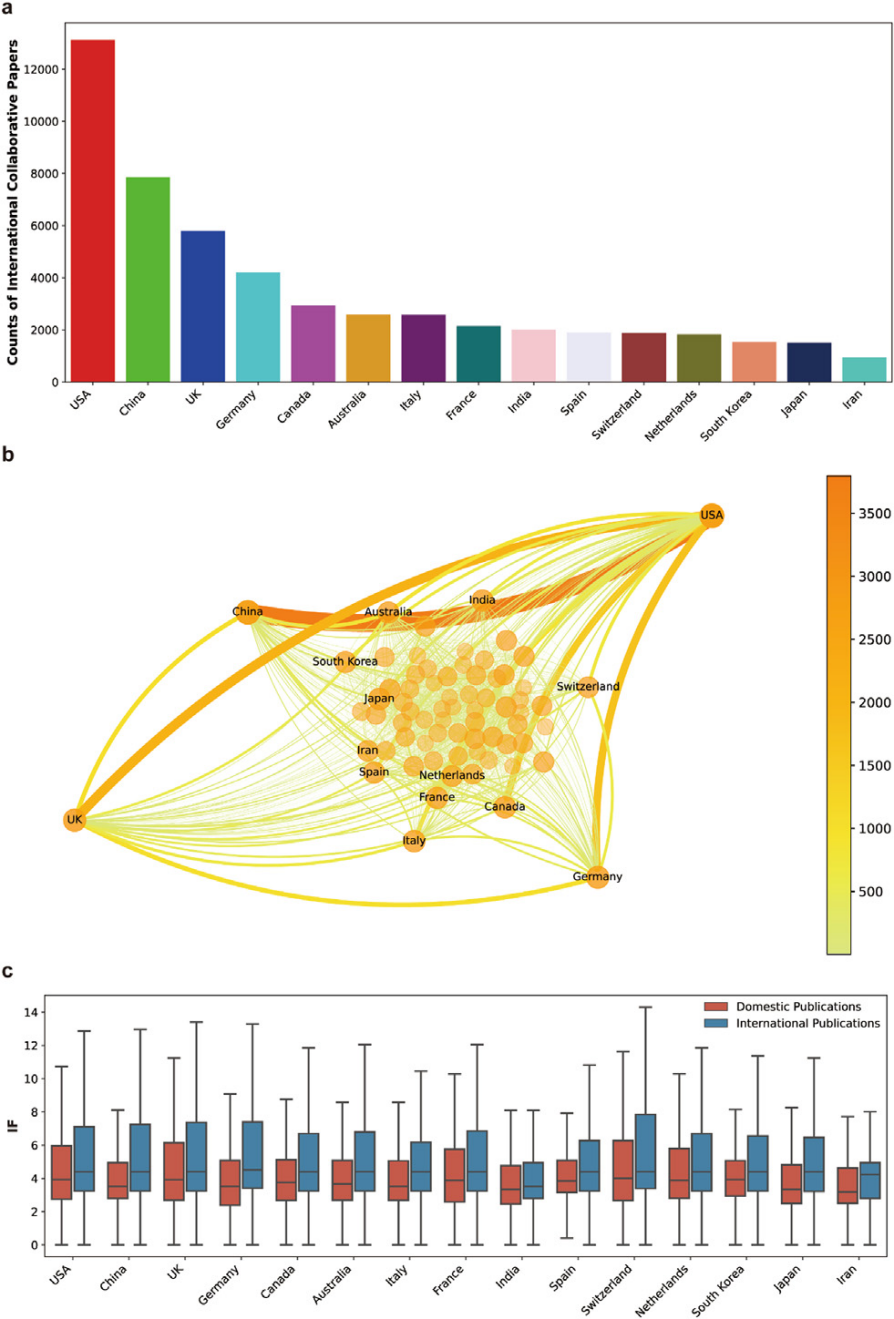
**International collaboration analysis of AI-related life science papers**. (a) List of the top 15 countries published the most international collaborative AI-related life science papers. (b) Country-wide collaboration network derived from AI-related life science papers. Each node represents a country, and the thickness of edges connecting two nodes is correlated with the amounts of collaborative publications produced by two countries. Only the top 15 countries shown in (a) are labeled. (c) Journal IF comparison between domestic and international publications produced by the 15 countries shown in (a). The bottom, middle, and upper lines of the boxes show the first quartile (Q1), median, and third quartile (Q3) of the journal IF of all qualified papers. Whiskers extended from the boxes represent values of Q1–1.5 × IQR (lower) and Q3 + 1.5 × IQR (upper).

We next analyzed the situation of international collaborations in AI-related life science research. Among the 139,799 analyzed papers, 29,104 were generated through international collaborations. Again, the USA led the international collaborative researches and generated the largest number of collaborative papers (Fig. 4a). The top 15 countries ranked by international collaborative AI-related life science publications are shown in Fig. 4a, countries following the USA are China, the UK, Germany and Canada. It is a bit surprising that although Japan hosts one of the most pioneer and famous international life science database, DNA Data Bank of Japan (DDBJ) [35,36], it only ranks the 14th in terms of international collaborative AI-related life science publications (Fig. 4a).

The international collaboration analysis diagram revealed that China and the USA had the strongest collaboration tie among all countries, evidenced by the largest number of publications produced by Sino-US collaborations (Fig. 4b). Large amount of joint publications were also produced by collaborations between the USA and the UK, as well as the USA and Germany (Fig. 4b). Compared with publications produced solely by domestic authors, papers generated by international collaborations tended to be published in higher impact journals, this phenomenon is consistent among the top 15 countries which were most active in international collaborations on AI-related life science research (Fig. 4c).

## 5 Analysis of major AI algorithms applied in life science research

**Fig. 5:**
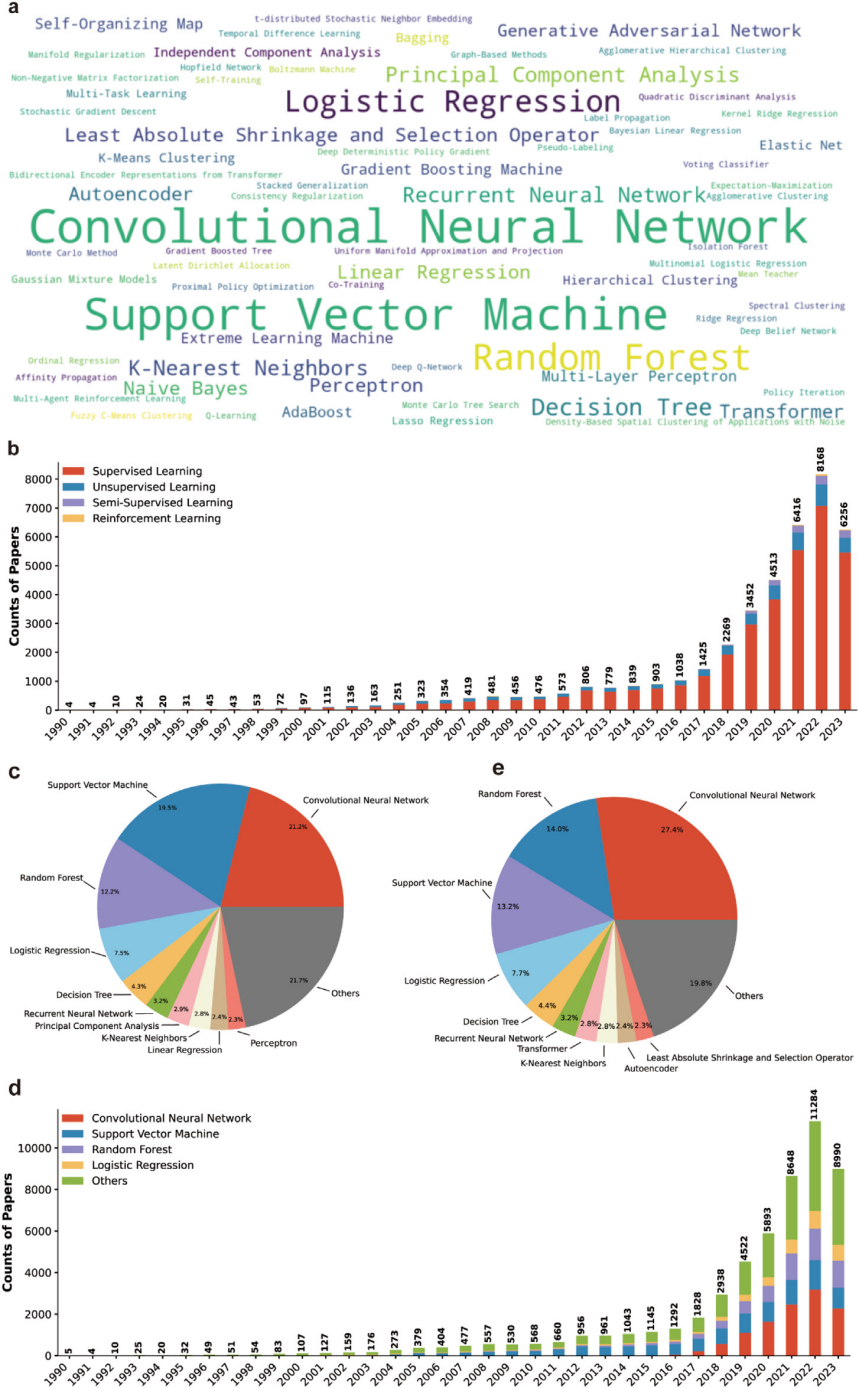
**Statistics of commonly used AI algorithms in AI-related life science research papers**. (a) Word cloud of the top 50 ranked AI algorithms used in AI-related life science researches by publication counts. (b) Annual counts of papers related to different categories of AI algorithms. (c) Frequency distribution of the top 10 ranked AI algorithms in AI-related life science papers by publication counts. (d) Annual counts of papers related to different AI algorithms. Only AI algorithms contributed to no less than 5% of AI-related life science papers are shown. (e) Frequency distribution of the top 10 ranked AI algorithms in AI-related life science papers since 2020 by publication counts.

To analyze what AI algorithms were used in life science research papers, we first generated a reference list comprising the names of 93 commonly used AI algorithms, then applied a combination of string matching and ChatGPT verification methods to identify the AI algorithms used in the literature we examined. A total of 38,826 papers including one or more AI algorithm names in their titles or abstracts were identified. Thus, the following statistical analysis only focused on these papers. We first generated a word cloud showing the relative frequency of the top 50 commonly used AI algorithms in the AI-related life science literature (Fig. 5a). The results showed that deep learning related algorithms (convolutional neural network, autoencoder, recurrent neural network, etc.) and classification algorithms (support vector machine, logistic regression, random forest, etc.) are widely used in life science research (Fig. 5a).

Following the common classification criteria, we grouped the identified AI algorithms into four machine learning categories, namely supervised learning, semi-supervised learning, unsupervised learning, and reinforcement learning. Supervised learning algorithms are the most widely used AI methods in life science research, accounted for 84.4% of the analyzed papers (Fig. 5b). Although unsupervised learning methods were applied in life science research about the same time as the supervised learning algorithms did, the proportion of papers they contributed gradually decreased since 2007 (Fig. 5b). The recently popularized semi-supervised learning and reinforcement learning related algorithms also gained increasing attention in life science research (Fig. 5b). For example, the semi-supervised learning algorithm Generative Adversarial Networks model was first applied in life science research in 2017 and showed a burst in publications since 2019, in accordance with the broad appreciation on the values of deep learning methods in scientific research.

In terms of individual AI algorithms, convolutional neural network, support vector machine, random forest, and logistic regression were the most commonly used methods, accounted for 21.2%, 19.5%, 12.2%, and 7.5% of AI-related life science papers, respectively (Fig. 5c). Other popular AI methods included decision tree, recurrent neural network, principal component analysis (PCA), K-nearest neighbor classification (KNN), linear regression and perception (Fig. 5c). It is worth to note that although convolutional neural network was ranked as the most popular AI algorithm in life science research, it only attracted researchers’ attention since 2015, and showed a rapid increment since 2019 (Fig. 5d).

To have a more detailed analysis on the impact of new AI methods in recent years, we specifically analyzed AI algorithms appeared in the titles or abstracts of life science papers published since 2020. Compared with the full timescale (1990–2023, Fig. 5c) analysis results, convolutional neural network was used more frequently since 2020 (Fig. 5e). Transformer, a revolutionized AI algorithm widely used in large language models, ranked the 7th among all AI methods used in life science research since 2020 (Fig. 5e), and is expected to be more widely used in the future. In addition, autoencoder, least absolute shrinkage and selection operator also emerged as popular AI algorithms since 2020 (Fig. 5e).

**Fig. 6:**
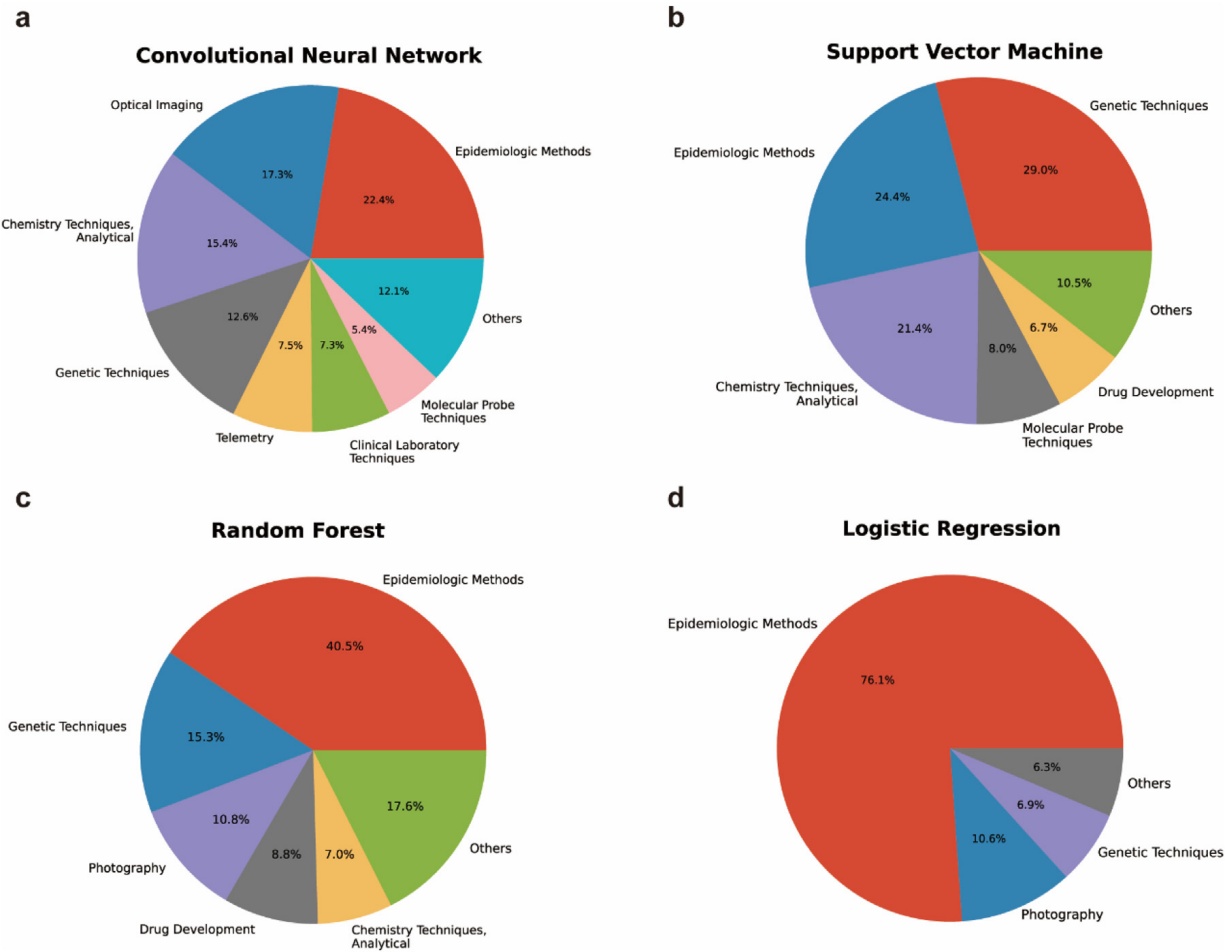
**High frequent MeSH term analysis under the “Investigative Techniques” category for popular AI algorithms**. (a-d) Frequency distribution of MeSH terms under the “Investigative Techniques” category for life science papers related to convolution neural network (a), support vector machine (b), random forest (c), and logistic regression algorithms (d), respectively. For each algorithm, only MeSH terms contributed to no less than 5% of total MeSH term counts from all qualified papers are shown.

We next used MeSH terms to analyze the research subjects of the top 4 most popular AI algorithms, namely convolution neural network, support vector machine, random forest, and logistic regression. MeSH terms are a set of vocabularies being assigned by the National Laboratory of Medicine (NLM) to each literature in the PubMed database to describe the paper's major content. To investigate the excelled application aspects of different AI algorithms, we collected MeSH terms under the “Investigative Techniques” category of all examined papers. Statistical analysis revealed that “Epidemiologic Methods” related MeSH terms were the top applications of convolutional neural network, random forest and logistic regression algorithms (Fig. 6). In particular, > 3/4 of logistic regression related papers were related to Epidemiologic Methods (Fig. 6). “Genetic Techniques” and “Chemical Techniques, Analytical” were also popular applications of these algorithms, although the contribution of “Chemical Techniques, Analytical” did not reach the threshold to be shown (Fig. 6). Support vector machine and random forest algorithms were more commonly used in drug development. In addition, random forest, together with logistic regression, were also popular algorithms in photography analysis (Fig. 6). Convolutional neural network, although only being widely recognized after 2017, has become the dominant AI algorithm for “Optical Imaging” and “Telemetry” related data analysis (Fig. 6a). It should be noted that the above-mentioned AI method popularity analysis were only conducted using the titles and abstracts of the selected papers, AI methods which are more frequently appeared in the main text or method sections of papers might be underestimated in this study.

## 6 Future perspectives on AI-assisted life science research

The above reviewed contents have shown the great power of AI in promoting life science research. It is evident that the field of life science research is one of the pioneers in generating big data and embracing AI methods, especially through the development and application of bioinformatics. On the other hand, the employment of AI methods, in combination with the big-data generation technologies, has transformed life science research into a new paradigm. Yet cautions still should be taken for better uses of AI in life science researches, especially from the following aspects. (1) To clearly define scientific questions suitable for AI to solve. The current AI models are mainly statistics based methods, therefore biological questions with enough training data and capturable features are more likely to obtain good results using AI methods. (2) Large AI models are not always better than traditional machine learning methods. Recently there has been evidence showing that large AI model may be more error-prone under certain circumstances [37], for some scientific questions, the traditional machine learning methods could be powerful enough or ever superior than large AI models. (3) To develop life science-oriented AI methods. Due to the high complexity of human and other organisms, as well as the lack of thorough data for most life science scenarios, the currently available AI methods are still not fully capable to reveal all underlying mechanisms and key factors for life regulation. To conquer these challenges, new AI models suitable for identifying unknown features from the complicated life science big data with multi-scale blurry features are in great needs.

Looking into the future, with the continuous development of AI methods and the accumulation of life science big data, AI-assisted life science research will usher in unprecedented opportunities. For example, the application of large language model has demonstrated great power in single-cell omics data analysis, biomarker identification, novel protein design and many healthcare related applications [[38], [39], [40]]. With the help of AI, scientists would be able to make more important discoveries, to accelerate drug development and disease treatment, and ultimately to uncover the underlying mystery of colorful lives. In the meanwhile, systems biology and synthetic biology would also be significantly benefited from AI-assisted researches, therefore to make greater contributions to human well-being and social development.

## Declaration of competing interest

The authors declare that they have no conflicts of interest in this work.
